# Magnesium in Aconitine‐Induced Electrical Storm—Mechanism Yes, Mandate No

**DOI:** 10.1111/anec.70133

**Published:** 2025-11-28

**Authors:** Zhong‐Qun Zhan, Hai‐Jun Xu

**Affiliations:** ^1^ Department of Cardiology Shenzhen Guangming District People's Hospital Shenzhen P. R. China

**Keywords:** aconitine poisoning, electrical storm, flecainide, magnesium sulfate

## Abstract

Aconitine poisoning produces lethal ventricular arrhythmias through persistent activation of cardiac sodium channels. We critically comment on a recent case report describing 28 biphasic shocks for aconitine‐induced electrical storm, addressing three fundamental questions. First, we demonstrate that verapamil is contraindicated as it worsens hypotension and reflex sympathetic activation without antagonizing sodium channel toxicity. Second, we review the evidence for magnesium sulfate: while basic science and observational data support its mechanistic role as a calcium antagonist and sodium channel modulator, Class IC antiarrhythmics (flecainide) demonstrate superior conversion rates (86% vs. 22% for magnesium monotherapy). Magnesium should be viewed as an evidence‐based adjunct, not mandated first‐line therapy. Third, we identify critical monitoring gaps including dynamic electrolyte assessment, ionized calcium repletion, and toxin quantification that must be addressed in future cases. We propose a practical, evidence‐based algorithm emphasizing: (1) first‐line flecainide 2 mg/kg IV, (2) magnesium sulfate adjunct targeting serum levels of 1.5–2.0 mmol/L, (3) aggressive calcium repletion to > 1.00 mmol/L, (4) early high‐flux hemoperfusion within 6 h, and (5) VA‐ECMO for refractory cases. Verapamil, diltiazem, and β‐blockers as sole agents should be explicitly avoided.

## Graphical abstract

Critical analysis reveals verapamil is contraindicated in aconitine poisoning and flecainide is superior to magnesium monotherapy. Our algorithm positions Class IC antiarrhythmics as first‐line, with magnesium serving as adjunctive metabolic therapy.

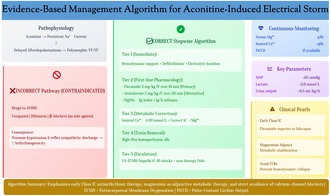

Ran et al. deserve congratulations for achieving neurologically intact survival in a 74‐year‐old man who received 28 biphasic shocks for aconitine‐induced electrical storm (Ran et al. 2025). Their report is a welcome addition to a sparse human literature, but it also exposes the persistent gap between bedside improvisation and evidence‐based toxicology. I will focus on three questions: (1) Was verapamil safe? (2) What does the evidence really say about magnesium? (3) How should we integrate these data into a practical algorithm?

## 1 Verapamil: A Wrong Drug At Any Dose

Aconitine binds to the neurotoxin‐binding site 2 of the cardiac voltage‐gated sodium channel Na_V_1.5, producing a persistent inward Na^+^ current, delayed after‐depolarizations, and triggered activity (Friese et al. 1997). Calcium‐channel blockers do not antagonize this mechanism; they depress nodal conduction, drop blood pressure and provoke reflex sympathetic discharge—an ideal setup for polymorphic VT. Chan's 14‐case series includes two patients who deteriorated within minutes after intravenous verapamil (Chan 2009). Coulson's systematic review lists verapamil and diltiazem as “contra‐indicated” in aconite poisoning (Coulson et al. 2017). The American Heart Association 2020 guidelines place Ca‐blockers in Class III (harm) for sodium‐channel‐toxic arrhythmias because they “may aggravate hypotension and provide no antagonism of sodium‐channel effects” (Panchal et al. 2020). The referring hospital's dose may have been small, but the choice was incorrect and should be explicitly discouraged in future protocols.

## 2 Magnesium: Mechanism Yes, Mandate No

### 2.1 Basic Science

In guinea‐pig papillary muscles exposed to aconitine, raising extracellular Mg^2+^ concentration from 1.2 to 4 mmol/L reduced the amplitude of delayed after‐depolarisations by 38% and abolished triggered ventricular ectopic beats through Ca^2+^‐antagonism and frequency‐dependent block of the fast Na^+^ current (Borchard et al. 1989). In vivo, a single 200 mg/kg Magnesium sulfate administered intraperitoneally doubles the VF threshold and prolongs survival in aconitine‐intoxicated rats (Liu et al. 2014).

### 2.2 Human Pharmacology

Intravenous magnesium sulfate 2 g achieves peak serum concentrations of ~1.5–2.5 mmol/L in patients with acute myocardial infarction, sufficient to reduce QT dispersion and inhibit L‐type Ca^2+^ current (Parikka et al. 1999). In a retrospective cohort of 36 severe aconitine‐poisoned patients, those given MgSO_4_ within 6 h required fewer shocks (median 3 vs. 8, *p* = 0.02) and had higher first‐shock success (78% vs. 38%, *p* = 0.01), although the evidence was graded B (limited) (Emergency Physician Branch of Chinese Medical Doctor Association et al. 2022). Coulson's systematic review identified nine patients receiving magnesium monotherapy; only two converted (22%), inferior to flecainide (6/7, 86%) or mexiletine (3/3, 100%) (Coulson et al. 2017). The present case illustrates salvage success after 28 shocks, not first‐line superiority.

## 3 Monitoring Gaps That Future Reports Must Close

Serum magnesium was not measured dynamically, so we cannot determine whether the patient was repleted or simply received a supra‐physiological dose. Ionized calcium fell to 0.96 mmol/L (lower reference limit 1.00 mmol/L); even mild hypocalcaemia can potentiate aconitine‐induced Na^+^ channel opening, yet no calcium replacement was documented (Friese et al. 1997; Emergency Physician Branch of Chinese Medical Doctor Association et al. 2022). Urinary or gastric liquid chromatography–tandem mass spectrometry screening could have confirmed exposure, but such tests are rarely available within the therapeutic time window. Continuous cardiac‐output monitoring (such as PiCCO) might have provided real‐time perfusion data, potentially reducing the trial‐and‐error phase of repeated shocks—this remains speculative but physiologically plausible.

## 4 Practical Algorithm (Evidence‐Based)

For haemodynamically unstable aconitine VT/VF:

- First‐line anti‐arrhythmic: Flecainide 2 mg/kg intravenously over 10 min (repeat once if needed); if unavailable, amiodarone 5 mg/kg over 20 min (Coulson et al. 2017).
- Concurrent electrolytes: Magnesium sulfate (MgSO₄, 2 g intravenous bolus followed by 1 g per hour infusion); target serum magnesium 1.5–2.0 mmol/L, measured every 4 h, halving the infusion rate if eGFR is below 30 mL per minute. Replete ionized calcium to > 1.00 mmol/L using 10% calcium gluconate (0.3 mL/kg intravenously over 10 min) when ionized calcium is ≤ 1.00 mmol/L or if haemodynamic compromise is present (Emergency Physician Branch of Chinese Medical Doctor Association et al. 2022).
- Early toxin removal: High‐flux hemoperfusion within 6 h (Class IIa, Level C); CRRT for multi‐organ dysfunction (Ke et al. 2024).
- Rescue extracorporeal life support: consider VA‐ECMO or Impella if more than ten appropriate shocks plus maximal medical therapy fail within 60 min, provided the center has extracorporeal life‐support capability (Coulson et al. 2017; Aziz et al. 2020).
- Avoid: verapamil, diltiazem, β‐blockers as sole agents, and further repetitive lidocaine boluses after adequate first‐line therapy (flecainide plus magnesium with electrolyte correction) has failed (Coulson et al. 2017; AHA 2020 guidelines on sodium‐channel‐blocker toxicity).

## 5 Research Priorities

Given the extreme rarity of aconitine poisoning, a traditional large‐scale RCT is unlikely to be feasible. Instead, we propose registry‐based target‐trial emulation with propensity scoring to estimate the marginal benefit of magnesium. Until such data are available, magnesium should remain an evidence‐based adjunct, not a substitute for early Class IC therapy.

## Author Contributions

Zhong‐Qun Zhan: Conceived the commentary, drafted the manuscript, critically revised it for intellectual content, and approved the final version. Guarantor of the work. Hai‐Jun Xu: Performed a comprehensive literature review, analyzed data from published studies, contributed to manuscript drafting, created the graphical abstract concept, and approved the final version. Both authors agree to be accountable for all aspects of the work and ensure that questions related to the accuracy or integrity of any part of the work are appropriately investigated and resolved.

## Funding

This work was supported by the Shenzhen Guangming District Program for Introducing High‐Level Medical Teams (szgmtd2025004).

## Conflicts of Interest

The authors declare no conflicts of interest.

## Data Availability

The data that support the findings of this study are openly available in figshare at https://doi.org/10.1111/anec.70133.
